# Correspondence—Board of Examiners

**Published:** 1881-05

**Authors:** E. G. Betty


### CORRESPONDENCE.

CINCINNATI, April 2d, 1881.

EDITOR REGISTER:—The matter I wish to communicate is not strictly within the province of Dentistry, and yet it is a subject in which both the profession and laity are deeply concerned.

It appears from the statistics of the Cincinnati Board of Health that the total number of births for the year 1880 amounted to 7945; of these 5623 were attended by midwives, the remainder, 2322, by physicians. The total number of still births for the same year was 344; the number of births attended, and the number occurring under the care of midwives was 124, while those in charge of physicians amounted to 220. The figures as here noted apparently indicate an inordinate proportion of still births under physicians' care, as compared with the total number occurring under the charge of the midwives. The discrepancy is accounted for by the fact that when the complications of delivery get beyond the ability of the midwife, a physician is called in and he then is obliged to make the death return at the Health Office. In this way the onus is thrown upon the physician, and under existing circumstances there is no remedy for him. It is proposed to request the State Legislature to pass a law regulating the practice of obstetrics by midwives in Hamilton County by establishing a Board of Examiners. This Board to consist of three members, two selected by the Cincinnati Obstetrical Society, these to act in conjunction with the Health Officer. All women engaging to practice midwifery after the passage of the law are to appear before this Board and undergo an examination to determine their competency. In addition, *all* midwives practicing in Hamilton County are to be registered at the Health Office, so that they will be held accountable.

Roughly estimated, the physicians attend but 35 per cent. of the total births. From this it is readily seen that nearly two-thirds of the births occurring in Cincinnati are in charge of incompetent persons.

The passage of such a law is greatly needed, and its enforcement is assured when the Health Officer, with his executive authority, is made a member of that Board. In fact, this matter is one requiring attention pretty much throughout the country in all the larger cities, and the Cincinnati Health Board is making a move in the right direction.

E. G. BETTY, D. D. S.

